# Psychometric Properties of the Korean Dispositional Hope Scale Using the Rasch Analysis in Stroke Patients

**DOI:** 10.1155/2019/7058415

**Published:** 2019-11-11

**Authors:** Eun-Young Park, Yoo Im Choi, Jung-Hee Kim

**Affiliations:** ^1^Department of Secondary Special Education, College of Education, Jeonju University, P.O. Box 560-759, 45 Baengma-gil, Wansan-gu, Jeonju, Republic of Korea; ^2^Department of Occupational Therapy, School of Medicine & Institute for Health Improvement, Wonkwang University, P.O. Box 54538, 460 Iksandae-ro, Iksan, Jeollabuk-do, Republic of Korea; ^3^Department of Clinical Nursing, College of Nursing, The Catholic University of Korea, 222 Banpo-daero, Seocho-gu, Seoul 06591, Republic of Korea

## Abstract

**Background:**

It is reported that hopeful thinking plays a positive role in encouraging patients to achieve functional goals during the rehabilitation process. Hope is a key concept in evaluating stroke outcomes in research and rehabilitation practice.

**Aims:**

The purpose of this study was to investigate the psychometric properties of the Korean Dispositional Hope Scale (K-DHS) using the Rasch analysis in patients with hemiplegic stroke.

**Methods:**

The K-DHS was completed by 166 community-dwelling hemiplegic stroke patients in Korea. Data were analyzed according to item fit, item difficulty, and the appropriateness of the rating scale using the Rasch analysis.

**Results:**

Item fit analysis showed that 8 items of the K-DHS are appropriate because the infit MSNQ was between 0.7 and 1.3. Item difficulty results revealed that there is a difference in distribution between personal attributes and item difficulty. It shows that the item fit statistics of the 4-point Likert scale of K-DHS are all good. The person separation index demonstrated that the K-DHS could differentiate two or three hope status strata in stroke patients. The item separation index indicated that the items were useful with high reliability.

**Conclusion:**

The K-DHS comprises appropriate items for measuring the hope of stroke patients living in the community, and the rating scale of the K-DHS is also appropriate. This study is the first to conduct an analysis of the rating scale and its appropriateness, as well as the difficulty of items based on item response theory, and offers new insights for enhancing hope and improving well-being following stroke.

## 1. Introduction

There is growing interest in positive psychology as part of a reflective concept against focusing only on the negative aspects of human nature [1]. Positive personality features (hope, optimism, spirituality, etc.) form a buffering action to protect the individual from the negative influences of risk factors such as life stress [2]. The most commonly used definition of hope in psychology is Bright et al.'s definition [3], “…a temperament, emphasizing the cognitive aspects of hope and the pursuit of goals.” Hope has positive correlations with academic achievement [3], the adaptive coping method [4], and flexible positive thinking [5]. Thus, people with high levels of hope are more capable of enduring physical pain [3] and show more rapid recovery from stress reactions than others [1]. Recent studies have reported that patients with high levels of hope showed better results in functional recovery, improved participation, and reduced depression in rehabilitation when compared to other patients [4, 5]. The effectiveness of interventions, such as positive thinking to improve self-concept and self-esteem, and social/family support to decrease depression has been reported [6, 7].

For stroke patients, one of the most important things is rehabilitation to maintain daily activities, and physical function, and then to recover an independent lifestyle. It is reported that hopeful thinking plays a positive role in encouraging patients to achieve functional goals during the rehabilitation process [3]. Due to physical impairment, a sense of hopelessness, and a fear of/anxiety about death [8, 9], stroke patients may also experience symptoms of depression [10]. For all these reasons, hope is a key concept in evaluating stroke outcomes in research and rehabilitation practice. The common concept of hope was Snyder's model [11]. Hope is conceptualized around three main components including goals, pathways, and agency. Specifically, goals defined as the process of thinking about one's goals, agency was along with the motivation to move toward those goals, and pathways were the ways to achieve those goals [12, 13].

One of the most commonly used assessment tools in evaluating hope is the Dispositional Hope Scale (DHS), which was developed based on the hope model proposed by Snyder et al. [14]. Although the DHS was developed to target undergraduate students, it is often used in other populations such as patients with mental disorders [15] and traumatic brain injury [16] as well as the general population. Also, the original DHS suggested a two-factor model which consists of two subscales measuring agency and pathways: agency is the motivational component for goal-directed determination, and pathway is a cognitive component for goal achievement [11]. Previous studies reported the dimensionality of DHS using factor analysis in university students [17] and students from 14 to 18 years of age [18]. Other studies reported the two factors of DHS in a multiethnic sample [19], Arabic individuals [20], injury survivors [21], and stroke patients [22]. Validity and reliability of DHS Japanese version was verified through confirmatory factor analysis and concurrent validity examination [23]. Recently, study for validation of the Malay version of DHS among 195 Malaysian cancer patients was performed and reported acceptable internal consistencies, test-retest reliability, convergent and discriminant validities and thus is suitable for assessing hope in Malaysian cancer patients [24]. However, there were differences in the structure of hope in stroke patients compared to the general population [22]. Also, some of the items such as “I've been pretty successful in life” has been loaded both factor and need for the new scale which would be argued [25]. Since no evidence regarding the usability of DHS especially for the parents with stroke, the question regarding its adequacy for use in different groups has still remained. Stroke patients have various challenges including maintaining activities of daily living, overcoming poststroke depression, and participating in rehabilitation training with a physical disability [26, 27]. Therefore, the psychometric properties of K-DHS among patients with stroke are likely to be different from the general population.

Rasch's modeling has many advantages when compared to the classic test theory approach. Item response theory, including the Rasch model, is a way to analyze the item by item characteristic curve unique to each item. The Rasch model has been recommended as a method for evaluating item fit and difficulty and is not affected by the characteristics of the subject group. The Rasch model has some strengths, including that it verifies the difficulty and discrimination power at each item unit and estimates the true ability of the participant on the basis of the analysis results. Also, the Rasch analysis has an advantage over item characteristic estimation [28]. It is logical to analyze each item when verifying the appropriateness of the tool, since the item is the most basic unit constituting the tool. Compared to classic test theory estimates, the ability of the subject, and the ability of the subject to be estimated from the total group and the total score of the test, the item response theory finds information on the subject's abilities and the characteristics of the test items themselves based on the subject's individual response to each item. Thus, item response theory is a systematic and logical method of verifying the appropriateness of the item [29].

Because the Rasch model differentiates redundant items or items with a low level of fit and determines whether each item covers the low level to the high level of the concept that we want to estimate, the Rasch model can determine whether to correct or delete the item based on stricter standards, as well as whether to raise the degree of item completion or verify the validity through confirmatory factor analysis. Therefore, in this study, through applying the Rasch model with the hope scale developed for targeting the general population, researchers in the current study assessed item difficulty, item fit, and rating scale fit and determined if they can apply to stroke patients.

The purpose of this study was to examine the psychometric properties of the Korean version of the DHS in stroke patients by using the Rasch analysis. In this way, the utility of the Korean version of the DHS can be determined with respect to patients with stroke.

## 2. Materials and Methods

### 2.1. Design and Participants

This methodological study used secondary analysis data measuring levels of hope by April 8, 2013 [29]. A total of 166 stroke patients living in the local community were included in the study. The study was approved (1041849-201311-BM-023-01) by the research ethics committee of Yonsei University. The subjects in this study were patients who had been diagnosed with stroke and achieved a score of more than 21 on the Korean version of the Mini-Mental State Examination [30]. There were 109 (65.7%) male patients and 57 (34.3%) female patients. The mean age of the subjects was 53.40 (SD = 13.47) years, and the average disease period was 48.42 (SD = 46.06) months. Cerebral hemorrhage and infarction were diagnosed in 78 patients (47.0%) and 88 patients (53.0%), respectively. Right-sided hemiplegia was present in 44.6% of patients, and left-sided hemiplegia was present in 55.4% of patients. Regarding education level, high school graduates were the most common educational level (*n* = 71, 42.8%) followed by college completion (*n* = 48, 28.9%). Middle school graduates were 27 (16.3%), and elementary school graduate were 17 (10.2%). The least frequent education level was no education experience (*n* = 3, 1.8%).

The current study utilized the DHS, developed by Snyder et al. [14] and verified in Korea, to measure hope levels in stroke patients. DHS is a scale for measuring hope using an individual's temperament and personal characteristics, and it consists of 12 items: 4 items assessing in each part (agency thinking, pathway thinking, and item for distractors). The items are answered on a four-point Likert scale, with a higher score indicating higher levels of hope. When DHS was developed, the item internal consistency was 74~.88 and test-retest reliability was *r* = .82 [31].

### 2.2. Data Analysis

Items were analyzed via the collected data through the Rasch analysis. The appropriateness of items was judged based on the infit mean square statistic (infit MNSQ) and outfit mean square statistic (outfit MNSQ). For the purposes of the current study, items with less than 0.7 infit MNSQ or more than 1.3 MNSQ are judged as inappropriate.

Regarding the comparison between personal attribute scores and difficulty, the analysis is conducted using the distribution of items and subjects. By transforming personal attribute scores and the difficulty of the items into logit scores and comparing them, the difficulty of the items and their appropriateness for the analysis group was evaluated. The questionnaire and the personality distribution chart were created by placing individual and question items on a graph according to their attribute scores and difficulty levels, respectively. A direct comparison was possible because the personal attribute score and the item difficulty were converted to the same logit scale, and it was possible to evaluate whether the item difficulty was appropriate for the group analyzed by direct comparison. The ranges of the two distributions agreed; that is, the difficulty of the item was similar to the range of the distribution so that it could measure the entire range of the individual property [32]. The estimates of the scale boundary points should be similar to the average ability estimates of the subjects, and the scale boundary points should also tend to increase as each scale score increases [33].

Rating scale analysis was conducted with the subject's score of each item, changes in the threshold estimates, and fit statistics. WINSTEP 3.6 [34] was used to analyze the Rasch model for items by each low level.

## 3. Results

### 3.1. Unidimensionality

Item fit analysis demonstrated that 8 items of K-DHS were appropriate because the infit MSNQ was between 0.7 and 1.3 (Table 1).

### 3.2. Item Difficulty

When comparing the personal attribute score and item difficulty, the 7^th^ item, “I've been pretty successful in life,” was the most difficult and the 3^rd^ item, “There are lots of ways around any problem,” was the easiest.

### 3.3. Rating Scale Analysis

The item fit of each scale score provides information for the function of the rating scale; item fit equal to and more than 1.4 indicates a malfunction (criteria: 1.0). The result shows that there is no inappropriate scale for K-DHS. It should be noted that the threshold estimate tends to increase as the scale score increases. The result shows that every low level of scale is proportional to the increase in scale score (Table 2).

### 3.4. Person and Item Separations

As provided in Table 3, the person separation was 2.76 and the reliability was 0.88 in person separation index. The item separation index was 4.53, and the item reliability was 0.95.

## 4. Discussion

The aim of this study was to examine the psychometric properties of the Korean version of the DHS in stroke patients using the Rasch analysis. This study applied the Rasch model to 166 stroke patients from K-DHS data and analyzed it according to test item fit and personal attribute scores and comparing the level of difficulty to the response categories to assess the adequacy.

Item fit aims to verify the unidimensionality of items and to estimate the MNSQ of item fit with a rating scale to determine how well-located each item is within the unidimensionality. A high MNSQ means that each item does not have different dimensions with other items, and a low MNSQ means that the item is redundant [32]. The appropriateness of items is judged based on the infit MNSQ and outfit MNSQ; an item that has less than 0.7 infit MNSQ or more than 1.3 MNSQ was judged as a misfit item for the purposes of this study. Infit value is more sensitive to the pattern of responses to items targeted on the person, and outfit value is more sensitive to responses to items with difficulty far from a person. Because outfit mean squares are influenced by outliers and easy to diagnose and remedy, it is less threatening to measurement. Infit MNSQ is influenced by response patterns and usually hard to diagnose and remedy, so infit MNSQ is a greater threat to measurement [35]. In this research, item analysis showed that all fit statistics for the eight items in K-DHS were good.

The Rasch analysis transforms item difficulty and the subject's ability into logit, and the conversion into these same units enables comparison of the item and subject on a single linear scale. An average measure is the estimate of average ability of the subject who answers the item; generally, as item scores increase, the estimates of average ability of subjects should also increase [35]. Results show that as the K-DHS score increased, the estimates of average ability of the subjects increased. Item difficulty results show that there was a difference in distribution between personal attributes and item difficulty. However, 38.6% of subjects are out of range for item difficulty in K-DHS, which means that K-DHS is problematic for evaluating subjects with very high or low level on the hope scale. The item “I've been pretty successful in life” is the most difficult, and the item “There are lots of ways around any problem” is the easiest. This means that one item was rarely reported among stroke patients with lower hope levels; the other item was frequently reported among most stroke patients.

Andrew et al. [36] reported that only 18% of stroke patients received all aspects of discharge care planning and these unmet needs could affect quality of life outcomes. Although stroke patients survive, they may still have physical disability or social withdrawal, such as loss of occupation [26, 37]. The stroke patients might be hardly item of “I've been pretty successful in life” among the DHS which were developed for general population.

There are persistent challenges among the stroke patients [27]. In the early periods of poststroke, feelings were stressful and uncontrollable and then returned to prestroke levels 12 to 18 months later [35]. In this adaptation process, self-efficacy among stroke patients can be enhanced over time [38, 39]. This may be the reason that the item “there are lots of ways around any problem” was easily agreed upon by stroke patients. The item is similar to the self-efficacy that is defined as the belief in one's capabilities to organize and execute the courses of action required to produce given attainments [40]. Also, considering the level of self-efficacy or self-control may change over time poststroke [41], further study should be conducted for investigating the progress of hope levels according to disability level and time.

The scale used in the test with rating scales also has clear categories such as latent variables. Fit statistics for each scale score give information as to whether the rating scale functions well or not. When the fit statistic for each scale score is equal to or more than 1.4 (criterion: 1.0), it implies that the scale does not function well, and gives information related to combining the scale score. It shows that item fit statistics for the 4-point scales are all good.

In terms of the person and item separation indexes, previous studies reported the reliability 0.88 of Cronbach's alpha in students from 14 to 18 years of age [18] and 0.826 in university students [17]. The separation index results indicated differences between person and item. Person separation index was lower than item separation index. Because person separation index was used to classify individuals, low person separation meant that the test might be separating the sample into enough levels. In general, person separation reliability of 0.9 means that the test could classify 3 or 4 levels, 0.8 could capture two or three levels, and 0.5 could capture one or two levels [42]. The 0.88 separation index of K-DHS differentiated two or three strata of hopeful thinking for stroke patients. High reliability of persons or items means that people with high ability are more likely to get high scores on measurement results. Considering that the item separation index indicated that the items were well spread with high reliability, the items of K-DHS could cover a wide range of hopeful thinking in stroke patients.

It has been reported that hopeful thinking may improve self-concept, self-esteem, and social/family support [6, 7] and the recovery of the functional level and health maintenance of stroke [4, 5]. Therefore, the level of hope among stroke patients should be assessed and evaluated in rehabilitation practice. This study could offer new insights for enhancing hope and improving well-being following stroke. Also, it could add to the emerging body of knowledge supporting that clinicians and researchers can use the K-DHS to assess hopeful thinking of stroke patients in clinical practice.

## 5. Conclusion

The K-DHS comprises appropriate items for measuring the hope of stroke patients living in the community, and the rating scale of the K-DHS is also appropriate. This study is the first to conduct an analysis of the rating scale and its appropriateness, as well as the difficulty of items based on item response theory, and offers new insights for enhancing hope and improving well-being following stroke. This study also had several limitations. Sample bias and self-reported bias might affect the interpretation of our data. Thus, the results of this study cannot be generalized to stroke patients who have significant cognitive deficits. Despite these limitations, the current study is the first to conduct analysis of rating scale, appropriateness, and difficulty of items through the Rasch analysis based on item response theory and determine the psychometric properties that are used when measuring the hope score of the stroke patients with K-DHS. Also, it could add to the emerging body of knowledge supporting that clinicians and researchers can use the K-DHS to assess hopeful thinking of stroke patients in clinical practice as well as the similar cultural background including Asian countries.

## Figures and Tables

**Table 1:** Item fit statistics: entry order.

Item	Measure	S.E.	Infit		Outfit	
			MNSQ	*Z* value	MNSQ	*Z* value
(1) I can think of many ways to get out of a jam.	0.34	0.19	0.79	-1.18	0.75	-1.90
(2) I energetically pursue my goals.	1.22	0.19	1.04	0.40	1.06	0.50
(3) There are lots of ways around any problem.	0.23	0.19	1.01	0.10	1.01	0.10
(4) I can think of many ways to get the things in life that are important to me.	0.30	0.19	0.83	-1.50	0.83	-1.40
(5) Even when others get discouraged, I know I can find a way to solve the problem.	0.23	0.19	0.95	-0.40	0.89	-0.70
(6) My past experiences have prepared me well for my future.	1.40	0.19	1.05	0.50	1.10	0.70
(7) I've been pretty successful in life.	2.95	0.19	1.28	2.10	1.34	2.00
(8) I meet the goals that I set for myself.	1.33	0.19	0.97	-0.20	0.94	-0.40

MNSQ: mean square, SE: standard error.

**Table 2:** Summary of the rating scale analysis of original 4-point scale.

Category level	Observed average	Infit MNSQ	Outfit MNSQ	Structure calibration
1	-4.68	1.00	0.98	None
2	-1.14	1.01	1.00	-4.85
3	1.70	0.99	0.99	0.08
4	4.81	0.95	0.97	4.76

**Table 3:** Person and item separation indexes.

Category	Person	Item
Separation index	2.76	4.53
Reliability	0.88	0.95

## Data Availability

The data used to support the findings of this study are available from the corresponding author upon request.
